# Political and environmental risks influence migration and human smuggling across the Mediterranean Sea

**DOI:** 10.1371/journal.pone.0236646

**Published:** 2020-07-31

**Authors:** Kara Ross Camarena, Sarah Claudy, Jijun Wang, Austin L. Wright

**Affiliations:** 1 Harris School of Public Policy, University of Chicago, Chicago, Illinois, United States of America; 2 US Department of the Navy, Washington, D.C., United States of America; ISDC - International Security and Development Center gGmbH, GERMANY

## Abstract

Since 2007 the number of refugees fleeing conflict and violence has doubled to more than 25 million. We leverage high frequency data on migration, sea conditions, and riots to investigate how political and environmental risks influence migration and human smuggling across the Mediterranean Sea. We report results from two observational studies. A high frequency time-series study demonstrates that risks alter migration patterns. An event study design demonstrates the effectiveness of a policy intervention that targeted Libyan militias engaged in human smuggling. The results highlight the important role of environmental and political risks in transit countries and their implications for migration and human smuggling.

## Introduction

In the last decade the number of refugees fleeing conflict and violence has doubled to more than 25 million. Between 2014 and 2019 nearly 2 million migrants crossed the Mediterranean. Some of these migrants qualify for asylum, and nearly all have used a smuggler [1]. Annually, human smuggling to the European Union (EU) is a 5 to 6 billion dollar industry [2], but crossing the Mediterranean Sea is risky. More than 18,000 people have died crossing to Europe since 2014. By 2016 the path from Libya to Italy by sea became the most traveled, and deadly, ‘clandestine’ route to Europe [3].

Initially, EU policy failed to stem the flow of migrants in the Central Mediterranean. Well into 2017, migrants arrived in Italy monthly, by the tens of thousands. However, in 2017 Italy began to address a new dimension of the problem: transit. EU policy had been aimed at stopping people from crossing the Mediterranean and addressing the so-called ‘root causes of migration’: violence, instability, and the failing economies of the migrants’ origin countries. Italy focused on shifting the incentives of those who facilitate transit across the Mediterranean. Our work shows that transit conditions influence arrivals in Italy, and the Italian intervention, which targeted militias that smuggled migrants, altered the transit environment and substantially slowed the flow of migrants across the Central Mediterranean.

Scholars are devoting greater attention to human smuggling [4–6]. Analysis of how smugglers and migrants strategically respond to enforcement at the frontier has also had a resurgence [7–9]. In particular, findings from along the US-Mexico border show that border enforcement, targeting migrants *but not smugglers*, leads to displacement along alternative routes [10]. Two complementary strands of literature focus on the process of migration. One examines the drivers of migration from countries of origin, highlighting violence, poverty, and environmental factors [11, 12]. The second explores transit in the Mediterranean region qualitatively. It describes the many steps migrants typically take in their journey and argues that the next leg of the journey is often not planned or envisioned until an opportunity arises [13, 14].

We contribute to this emerging body of work by analyzing migration through transit countries quantitatively. We choose the word ‘transit’ to link our work the qualitative work on ‘transit migration.’ To our knowledge, our study is the first quantitative study of how environmental and political conditions in transit countries influence migration and human smuggling patterns. We examine migrants’ responses to competing risks in transit. Then we evaluate the effectiveness of a policy intervention targeted at the militias that smuggle migrants. Our analysis complements work on border enforcement by establishing ways that the transit environment drives migrant flows. Our work also complements qualitative research on transit and the importance of reputation and relational contracting within smuggling markets [5, 15, 16].

With detailed data about crossings from North Africa to Italy, we show a robust relationship between migrant arrivals in Italy and political unrest in North Africa. A 10 percent increase in riots near ports where migrants congregate corresponds to a 4.89 percent increase in migrants arriving in Italy. A reluctance to leave in risky sea conditions drives arrivals too. A 10 percent increase in wave height corresponds to a 27 percent decrease in arrivals in Italy. These findings are robust to a number of alternative model and outcome specifications.

We examine the importance of militias, that smuggle migrants, using an event study motivated by an Italian-led intervention. In mid-2017, Italy, with financial support from the EU, aimed to disrupt migration by co-opting militias that facilitate smuggling and giving them access to money as coast guard. We find that 343 fewer migrants arrive in Italy per day, following the intervention, a 0.6 standard deviation reduction in migrant crossings. The reduction occurs despite little change in demand. Underlying drivers of migration from origin countries in Sub-Saharan Africa changed little in 2017, and the number of international migrants in Libya remained stable. Further, there is limited evidence of displacement of migrants to alternative routes and no evidence of increased death rates at sea.

Our findings suggest transit conditions are important for understanding migrant flows. Violence not only drives migrants from their countries of origin, violence in transit countries may create pressure to move along the route toward a migrant’s next destination. Environmental risks during travel reduce migrant flows. Evidence from the Italian intervention in Libya suggests non-state actors within transit countries were used effectively to stem the flow of migrants.

## Materials and methods

### Context and data

The human smuggling market in North African port cities is competitive [5]. When smugglers began using inflatable craft that are easily replaced, entry barriers were further reduced. The price for crossing the Mediterranean fell substantially. From the initial surge in 2015 to 2016, prices for the sea leg of the journey were 200 to 250 USD. In June 2017, prices were as low as 60 USD. Following the intervention we study, qualitative assessments suggest prices rebounded [17].

By 2014 when smuggling across the Mediterranean was expanding rapidly, there was little government infrastructure to curtail it. The Libyan Navy and Coast Guard’s vessels were among the targets of the 2011 NATO intervention in Libya. Militias and other armed groups controlled ports and the surrounding water. In the context of a thriving smuggling industry, militias smuggled migrants themselves, charged tolls to other smugglers for passage out of controlled waters, and operated detention centers for migrants who were captured by formal and informal authorities [18].

The journey from departure in Libya or Tunisia to arrival in Italy takes from one to seven days. In a seaworthy boat, the trip takes less than a day [19]. However a journey in a wooden fishing boat or rubber dinghy can take several days. The most detailed estimates of trip duration are from 2013, before there were NGO rescue boats; they suggest smuggled migrants’ trips were 2 to 6 days [20]. This variation is mostly due to time at sea before rescue [20]. During the period we study, NGO boats are rescuing a substantial portion of migrants, and migrants are traveling in their rubber dinghies shorter distances than in the 2013-14 period [21]. Taken together, the qualitative evidence suggests that most journeys take two to three days with some travel taking seven days or more.

To account for this uncertainty in how long cross-sea trips take, we use a measure of sea and political conditions that leverages variation from the seven days prior to arrival. This choice relies on reports from extensive interviewing of migrants upon their arrival in Italy and some smugglers in Libya. Typically, when a migrant in Libya decides to go to Europe, the first step is to contact an intermediary. The intermediary takes the migrant to a holding place, where migrants wait until sea conditions are favorable and there are enough people to fill a boat [22]. Once there are sufficient numbers (80-100), the migrants board a rubber dinghy, and one migrant is charged with piloting the boat for 4-8 hours straight into international waters. Then, the migrants make an SOS call with an included radio [22] and wait to be rescued. Once on a rescue boat, they make their way to a close, designated port. Our arrival data is a composition of estimates from the United Nations High Commissioner for Refugees (UNHCR) and registration from the Italian Ministry of the Interior. These numbers are collected the day of arrival, or within the next 2 days, depending on the port and the timing of UNHCR access to arriving migrants [23]. Econometrically, using variation from a longer lag introduces some noise since the additional lags are only relevant to a (potentially small) subset of migrants. This would cause attenuation bias. If true, this type of bias would lead us to underestimate the true effect (i.e., what we would estimate if we had data on the duration of each individual’s journey).

**Fig 1:**
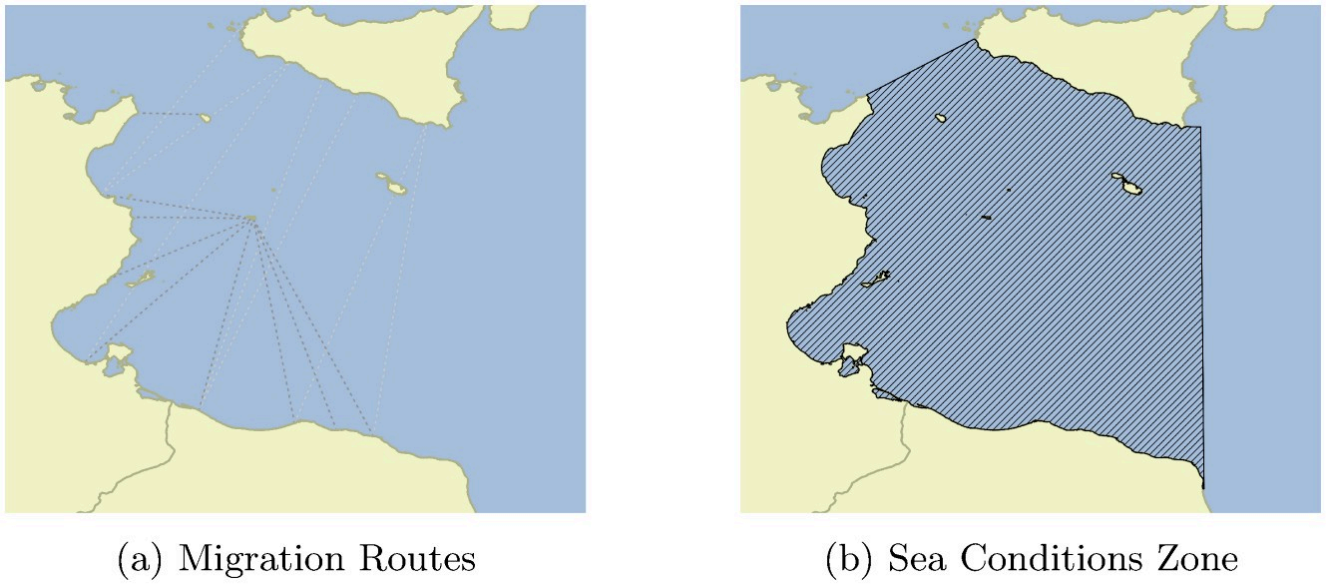
Region of Study migration passages and smuggling hubs (a) and area used for calculating sea conditions (b). Map of smuggling routes is recreated based on information from the IOM [24]. Sea condition zone is authors’ projection. Data on sea conditions is from the Copernicus marine environment monitoring service. Boundary files are from GADM [25].

We study migrant movement into Italy from January 2016 to April 2018. Our data tracks the arrival of 307,056 individuals and comes from UNHCR, through the Operational Portal—Mediterranean. The arrivals were largely processed at the Lampedusa Reception Center, located in the Italian Pelagie Islands. Remaining counts were compiled at other facilities along the Italian coast. These records do not distinguish origins, yielding a daily time series of migration. For more information on origins, we use routes based on qualitative research by the International Organization for Migration (IOM). There are nine primary sea routes from ports in Libya and Tunisia to Lampedusa (see Fig 1).

Migrants’ transit may be influenced by political unrest. To capture variation in political unrest near areas where migrants depart, we construct 25-kilometer buffers around these nine ports. We identify all riots within these buffers collected in the Armed Conflict Location & Event Data Project (ACLED) [26]. We select riots because they occur in urban areas and are more likely to affect labor migrants than organized violence. We expect migrants to respond to riots because they make an area less safe and may be indicative of more systematic violence to come. We anticipate that migrants should be less affected by violence targeting civilians and rebel violence, as migrants may avoid places that are targets of a government crackdown or have ongoing rebel violence. During our sample period, nearly all events recorded within our buffers were georeferenced to the town (spatial precision level 1), giving us more confidence in the assignment of riot events and other instances of violence to ports.

Because sea travel takes up to one week from departure to registration of arrival, we count the number of riot events in the prior week. For migrants with long journeys (6 to 7 days), this implies that there is noise in this measure (since future riots are difficult to anticipate and likely do not effect smuggling). This noise likely causes attenuation in the estimated relationship with riots. We investigate this in the Robustness section below. We follow a similar strategy for investigating violence against civilians and rebel-government violence (S2 Table). We find no consistent evidence that these other forms of violence influence migration as much as riots, though the estimated effects of civilian harm are imprecise. For this reason, our main analysis focuses on riot events.

Migration may also be influenced by sea conditions. To account for this, we gather daily sea wave height data from the EU’s Copernicus marine environment monitoring service [27]. Wave heights were recorded at 0500 local time (approximately sunrise). We calculate wave conditions within a geographic zone derived from the IOM sea routes, enabling us to capture wave heights along and around these passageways (see Fig 1). Similar to our riot data, we construct an average of wave heights from the prior week to account for the lag between departure conditions and arrivals in Italy. For migrants with short journeys (1 to 2 days), this implies the link between migration and wave height will be attenuated (since migration is unlikely to be influenced by historical wave height conditional on current wave conditions). We assess these dynamics below in the Robustness section.

We supplement arrival records with data from the IOM’s Missing Migrants Project [28]. These data are clustered by event and provide estimates of the number of migrants killed or missing. These records include migrant deaths from boats capsizing and more regular occurrences like deaths at sea due to dehydration, illness, or injury. Coordinates are associated with the death if known and otherwise, the place of recovery. The Missing Migrants Project gathers data from official sources like medical examiners and coast guard. They also draw on data from media sources and NGOs that help migrants and their families. The IOM platform is the most comprehensive available and is used by governmental and non-governmental actors for tracking casualties. We collapse these records into a daily time series and, as a robustness check, combine our information on arrivals with this data to calculate a daily total flow (of those who did and did not arrive in Italy).

### Time series analysis: Political and environmental risks

We anticipate a tradeoff between political risks (e.g., exposure to riots) and environmental conditions which increase the probability of death at sea (dangerous wave conditions). We exploit the daily time series data above to study this question. Our main specification incorporates political unrest (riots) and sea conditions. We begin by estimating Eq 1:

$$\begin{array}{c} arrivals_{i}=\alpha +\beta_{1}riots_{i}+\beta_{2}wave\_height_{i}+\gamma_{}month_{i}+\lambda_{}day_{i}+\epsilon \end{array}$$ (1)

Where *arrivals*_*i*_ is the daily total of arrivals, *riots*_*i*_ indicates the intensity of riots in the prior week, and *wave*_*height*_*i*_ captures average sea conditions in the prior week. To ease interpretation as elasticities (percentages), we evaluate the log of these three measures. For consistency, we add one to *arrivals*_*i*_ and *riots*_*i*_ before calculating the logarithm. Supplemental models incorporate *month*_*i*_ and *day*_*i*_. *month*_*i*_ indicates month of year fixed effects, and *day*_*i*_ represents day of week fixed effects. We leverage *month*_*i*_ to capture any seasonal trends in flight, violence, or sea conditions. *day*_*i*_ helps us to account for any systematic variation in arrivals that might be driven by intake operations (e.g., the facility may staff fewer officers on Sundays). In our main specification, we produce heteroskedasticity robust standard errors. To account for the possibility that flows are serially correlated over time, we also produce Driscoll-Kraay temporal autocorrelation robust standard errors.

Migrants may leave Libya in response to riots; however, it is less clear whether the smugglers or the migrants are the decision makers with respect to rough seas. Qualitative evidence suggests that it is the smugglers who decide when the boats leave. Frontex, the European Border Agency, explains that, “Many people who are coming from Africa have never seen the sea in their lives before,” [21, par. 18] which makes it unlikely the migrants are accurately judging the seas. Based on primary data collection in Libya, UNHCR contract researchers concluded that migrants wait “until the smuggler decides that the conditions are right for travel” [22, p. 144]. Economic theory provides a rationale for why smugglers have long term incentives to get their migrants into Europe. Specifically, Mediterranean human smuggling is built on repeated interactions [15]. With repeated interactions smugglers and intermediaries build reputations similar to firms with relational contracts in supply chains [29, 30]. This intuition is similar to Campana 2018 [5], which studies networks of smugglers in Libya. In part, the agency of the smugglers is the impetus for examining the Italian intervention.

### Event study: Italy’s 2017 Libya intervention

We leverage an Italian policy intervention backed by EU funding to examine the role of Libyan militias in human smuggling across the Mediterranean. The Italian policy is aimed at disrupting the human smuggling business in Libya, while doing nothing explicitly to address the flow of migrants seeking to travel across the Mediterranean. An important discrete change in July 2017 allows us to examine the role of the militias involved in human smuggling: Italy coordinated with militias engaged in human smuggling and incorporated them into an “anti-smuggling” coast guard force.

At the end of June 2017, Italy facilitated a deal with militias in the key port of Sabratha, west of the capital, Tripoli. The deal turned militias that facilitated smuggling into an “anti-smuggling” coast guard [18, 31]. The deal also gave the militias access to funds and equipment for coast guard activities. Italy brokered a similar agreement in Southern Libya that built a coalition among Southern tribes and the UN-backed government in Libya and gave them access to EU funds [32]. While the Italian Foreign Minister acknowledged Italy “spoke to everyone,” the Italian government has not acknowledged its part in the Sabratha deal [33, 34]. By the beginning of July, the Libyan Coast Guard had refurbished boats, and joint operations between the EU-bolstered Libyan Coast Guard and Italian Navy ensured that 60% of intercepted migrant boats were being returned to Libya [18, 34]. At the same time, Italy announced a draft code of conduct that placed restrictions on NGO rescue ships [18]. Subsequent investigations suggest that the Libyan government used the cash influx from the EU to pay off or co-opt militias who controlled ports, acted as coast guard, and facilitated migrant smuggling and detention [17]. The Italian intervention is ideal for estimation because migrants could not have anticipated the policy. The median migrant spends 6 to 12 months in Libya (based on the IOM Displacement Tracking Matrix) and documentary evidence has Libya turning around most boat within 2 weeks.

Targeting militias in the Central Mediterranean may be particularly important. Libya to Italy sea crossings are distinct among irregular transit because migrants typically use a smuggler [1]. Migrants crossing land borders often make the crossing on their own. Particularly, circular migrants who cross regularly know the way and know how to avoid border enforcement. Those who are less experienced or wish to reduce risk choose to use a smuggler [1, 6]. Because crossing the Central Mediterranean is so difficult, almost all irregular migrants engage a smuggler, which presents an opportunity for Libyan militias to profit.

This intervention differed from other interventions pursued by the EU and Italy. Beyond changing the cost of migrating or the expected utility of moving to Europe, it altered the supply side of the smuggling market. Militias in Sabratha stopped smuggling migrants and began operating as a coast guard force. The intervention, thus, operated through two channels. Like other interventions it decreased the probability a migrant arrived in Europe. The intervention also reduced the number of illicit organizations engaged in smuggling. The next component of our analysis focuses on the impact of the Italian intervention on migrant arrivals.

Italy received orders of magnitude more migrants than any other country in Europe during the period studied. Flows into other European countries therefore cannot serve as reasonable counterfactual trends. Because we lack a viable control group, we implement a synthetic prediction model (using high dimensional time fixed effects) to construct a counterfactual flow of migrants for 2017 based on 2016 migration patterns (the only complete migration season we observe before the intervention). We use calendar-week specific parameters to capture the seasonal trend in migration from 2016. Using this approach to estimate the counterfactual trend enables us to be agnostic about functional form of the seasonal trend. Rather than relying on a linear or higher order polynomial approximation of the trend, we estimate 52 separate slopes. This high dimensional estimation captures the underlying seasonal trend more precisely. We use the 2016 seasonal trend to construct a high resolution counterfactual trend for 2017. We then remove the constructed trend from the realized trend in 2017. This residual measure, our dependent variable in the event study, captures the variation in 2017 that is unexplained by high dimensional seasonal variation in the prior year. The timing of the intervention sets up an ideal shock from which we can estimate a regression model analogous to a difference-in-differences approach using Eq 2:

$$\begin{array}{c} arrivals_{i}^{ds}=\alpha +\beta_{1}Post\_Intervention_{i}+\epsilon \end{array}$$ (2)

Where $arrivals_{i}^{ds}$ is the weekly mean of arrivals (deseasonalized) and *Post*_*Intervention*_*i*_ is the quantity of interest, the change in migrant flows into Italy following the intervention. The coefficient on *Post*_*Intervention*_*i*_ captures the deviation after the intervention from the seasonal migration trend (when no comparable intervention took place). This deviation is equivalent to the level difference in a difference-in-differences research design. Following our most conservative time series specification, we produce Driscoll-Kraay temporal autocorrelation robust standard errors (with a bandwidth of four weeks).

## Results

### Political and environmental risks

Riots in North Africa are positively correlated with migrant arrivals in Italy, and higher wave heights decrease these arrivals. Table 1 reports the results from our main specifications in Eq 1. Summary statistics are presented in S1 Table. Our interpretation of the main effects is consistent with an elasticity between our independent and dependent variables. In Column 1, we present the baseline correlation between arrivals and political unrest in the prior week. We find evidence consistent with our expectation, a strong positive relationship. A 10 percent increase in riot intensity corresponds to a 4.89 percent increase in arrivals. In Column 2, we introduce our measure of travel risk. If our argument is correct, we would expect a negative correlation between wave height and arrival intensity. Here, the evidence is even sharper. A 10 percent increase in wave heights leads to an approximately 27 percent decrease in arrivals. It is possible that sea conditions are linked to riot activity. Jointly estimating these relationships in Column 3 allows us to partial out any residual pair-wise correlation. Our estimates are stable. In Column 4, we introduce *month*_*i*_ fixed effects to the model, which helps to account for seasonal variation in migration, violence, and sea conditions. Our estimate of the impact of sea risks is marginally attenuated, but remains more than five times larger in magnitude than political unrest. It is possible that staffing schedules or intake regulations lead to higher levels of registered arrivals on certain days of the week. To account for this, we add *day*_*i*_ fixed effects to Column 5. Our results are unaffected. It is also possible that the intensity of arrivals, and other conditions, may be strongly correlated over time. If so, our baseline approach to standard errors might overstate the precision of our estimates. Thus, we calculate Driscoll-Kraay temporal autocorrelation robust standard errors in Column 6 using a 14 day window, which we believe is conservative. The precision of our estimates decreases, but the parameters of interest, riot intensity and wave height, remain correlated with migration patterns. In S3 Table we demonstrate that our inferences are consistent for 21, 28, and 56 day bandwidths. We also present results for clustering by month. All results are substantively consistent and precise at conventional levels.

**Table 1:** Riots, sea conditions, and migrant flows to Italy.

	(1)	(2)	(3)	(4)	(5)	(6)	(7)
Riots (ln, prior week total)	0.489***		0.478***	0.503***	0.503***	0.503**	0.467**
	(0.174)		(0.162)	(0.163)	(0.164)	(0.200)	(0.197)
Wave Height (ln, prior week average)		-2.715***	-2.711***	-2.542***	-2.542***	-2.542***	-2.259***
		(0.233)	(0.231)	(0.333)	(0.332)	(0.364)	(0.352)
Number of Observations	812	812	812	812	812	812	812
R^2^	0.00914	0.130	0.139	0.171	0.174	0.0802	0.0742

Outcome is the daily total of migrants arriving in Italy (ln) (Columns 1-6). In Column 7, the outcome is the daily total flow of migrants (the sum of arrivals and reported deaths and disappearances). Total number of riots in the prior week (ln) and average sea conditions (wave height) during the prior week (ln) are the regressors of interest. Unit of analysis is the day. Heteroskedasticity robust standard errors are reported in Columns 1-5; Driscoll-Kraay temporal autocorrelation robust standard errors (clustered by 14 day windows) are reported in Column 6-7. The R^2^ reported in Columns 6-7 is the within fixed effect variation explained by sea conditions and riots, which is why it differs in magnitude relative to 1-5, which report the standard measure. Stars indicate

*** *p* < 0.01,

** *p* < 0.05,

* *p* < 0.1.

Another possibility is that the patterns we observe are driven by survivor bias: during poor sea conditions, fewer migrants survive and are able to make landfall. The negative correlation between risky sea conditions and arrivals, therefore, may be biased in the direction of our argument, overstating the elasticity. If, instead of arrivals, we were able to identify the total flow of migrants, it would address this concern. That would involve combining arrivals with information on the flow of migrants that die or disappear (presumed dead) on their sea passage. To investigate this, we rely on the IOM’s data on missing migrants. We collapse this data into a comparable daily time series and sum arrivals and deaths from this source into a total migrant flow. We replicate our most conservative model specification in Column 7 with this new outcome. The sea conditions coefficient decreases in magnitude, from 2.54 to 2.26. The riots coefficient also declines, which is consistent in relative scale. This suggests that our benchmark results are marginally influenced by survivor bias.

**Fig 2:**
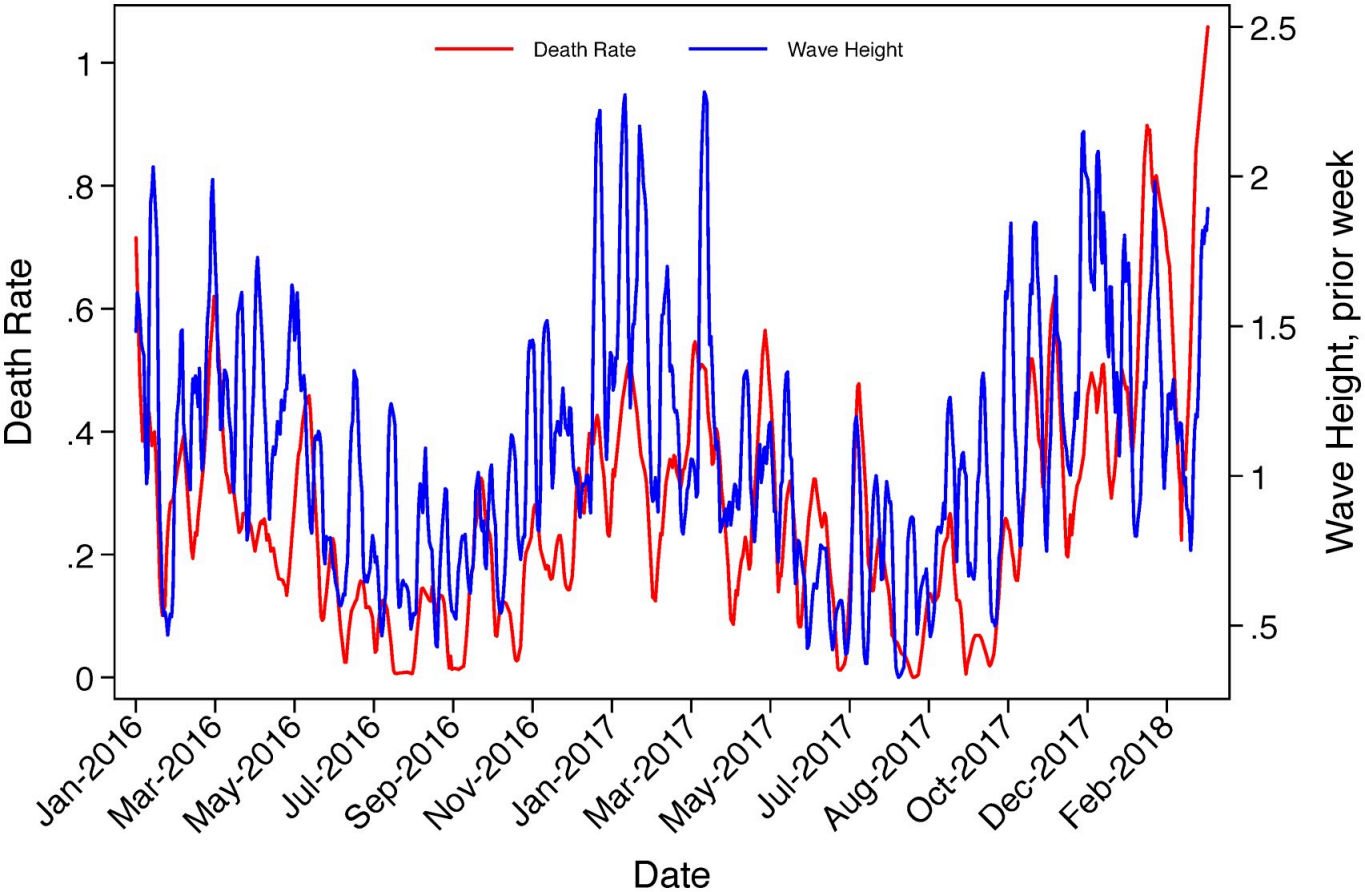
Trends in death rates and Sea conditions during sample period. Daily death rates are calculated using IOM data on migrant deaths and disappearances in the Mediterranean and UNHCR intake data for the Italian coast. Wave heights are drawn from the Copernicus platform.

Our data on migrant casualties allow us to verify the mechanism of our argument, that poor sea conditions increase the risk of death. We indeed find higher waves correspond to a higher death rate. To evaluate this mechanism, we calculate a daily death rate. Because this rate is unobserved if migrants neither arrive nor die on a given day, our design is now an interrupted daily time series. We begin by visualizing this relationship in Fig 2. The two trends, death rates and wave heights, covary closely. As waves reach dangerous levels (greater than 1.5 meters for most rubber craft), death rates increase substantially. We next present statistical evidence of the correlation between the death rate and sea conditions in S4 Table, following the main specification in Column 6 of Table 1. We focus first on the correlation between the current death rate and current wave height (Column 1). We explore the effect of the first lag in Column 2. This allows us to account for the possibility that there is a lag between reported death events and the precipitating weather event. The effect of the first lag relative to the day-of measure is large and statistically distinct. In Column 3, we attempt to capture the effect of an extended set of wave height lags; we find compelling evidence that the first lag explains variation in death rates, though the joint significance of the second and third lags is also statistically different from zero (*p* = 0.0280). This is reflected in Column 4, where we use a three day moving average of wave height and find robust and substantively large effects. For consistency with Table 1, we also demonstrate that the moving average of the week prior to the death event is highly significant and large in magnitude. These estimates confirm that there is a strong positive relationship between sea risk and the percentage of migrants who die or are lost at sea.

### Targeted intervention

**Fig 3:**
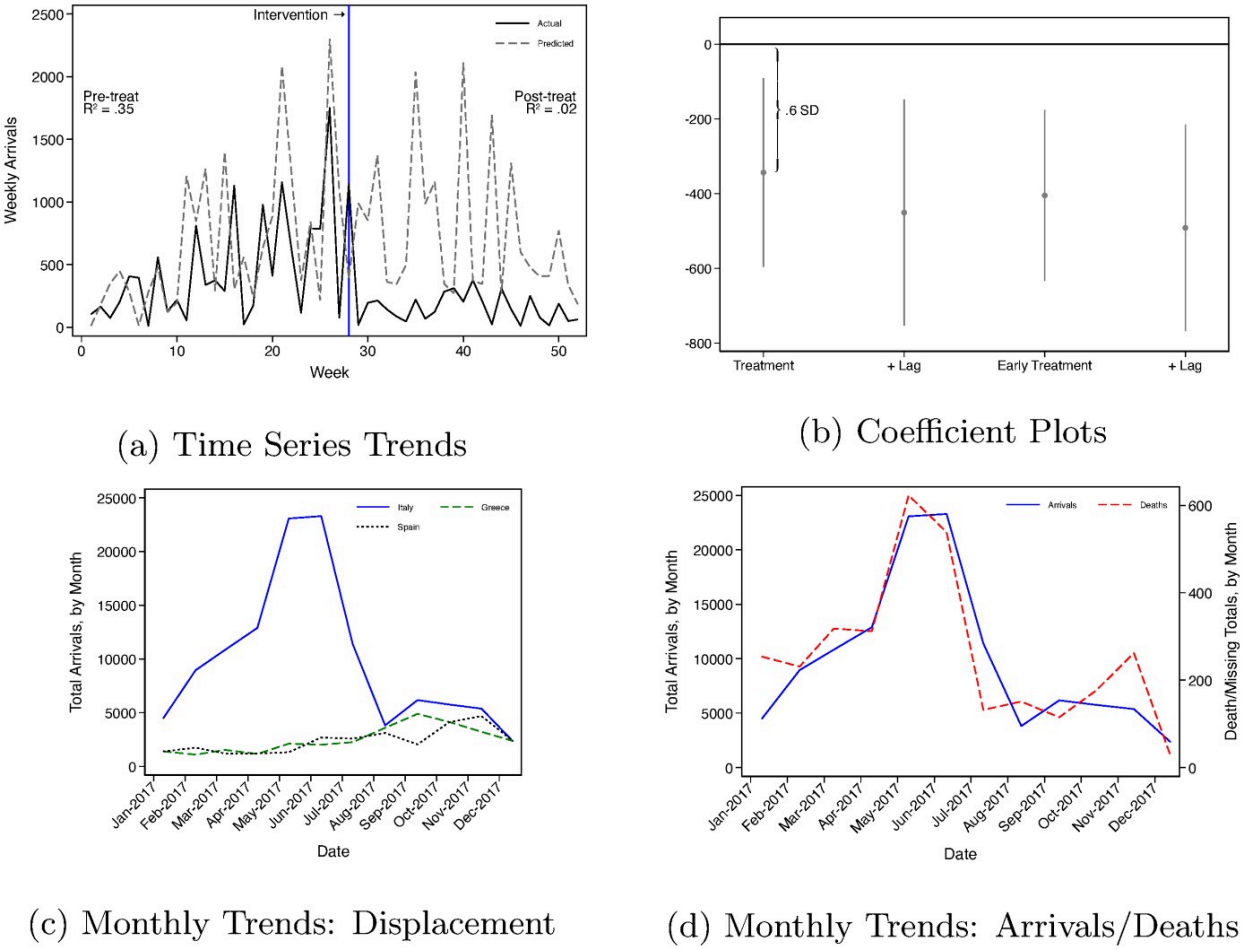
Impact of Italian intervention. (a) Weekly time series of actual (solid) and predicted (dashed) arrivals. Blue vertical bar indicates the week of the intervention. As expected, fit statistics suggest the predicted variation maps well to the pre-treatment period and poorly to the post-treatment period. (b) Regression-based estimates of the reduction in migrant flows following the intervention, relative the predicted flows from the prior year. Reduction is equivalent to 0.6 standard deviations. Outcome mean is -387.1; standard deviation is 595.2. 95% confidence intervals shown. (c) Monthly trends in migrant flows to three receiving countries. Notice large reduction corresponds to minor displacement through Spain and Greece. (d) Monthly trends in arrivals and deaths. Note strong correspondence between two trends (R^2^ = .79).

Fig 3 displays the results of the Italian intervention study. It shows that the intervention reduced migrant flows. Plot (a) traces weekly arrivals in Italy during 2017 (solid line) and the predicted weekly arrivals in Italy using 2016 arrivals to predict 2017 arrivals (dashed line). Prior to the policy intervention, counterfactual predictions based on 2016 weekly arrivals explain 35% of the variation in arrivals in 2017. Migrants flows are highly seasonal, and one year’s arrivals are a good predictor of the next. Italy’s intervention occurs in mid-July, and the drop in arrivals is visible. Following the intervention, the 2016 migrant flows explain only 2% of the variation in arrivals. Plot (b) displays estimated coefficients from the deseasonalized estimation approach in Eq 2. The first point plotted is our preferred specification which marks the intervention at Week 28, the earliest week that we could document the Libyan Coast Guard turning around most boats. We estimate the the policy intervention reduced migrant arrivals by 343 people per day for the remainder of 2017. As robustness checks, we include estimates from three other specifications. The second estimate includes controls for the average number of daily arrivals in the previous week. The third estimate uses an earlier week for the intervention, Week 26 which follows the weekend when Italy is reported to have negotiated with armed groups in the port city of Sabratha. The final estimate uses the earlier treatment week and includes lagged arrivals. Regardless of the specification, the estimates are stable and statistically significant at conventional levels.

It is possible that Italy’s intervention successfully limited flows into Italy while causing other problems; we consider two. First, it may be that when crossing from Libya to Italy became too difficult or costly, migrants moved through alternative routes. The two most natural routes are through the Western Mediterranean into Spain or through the Eastern Mediterranean into Greece. Panel (c) shows that there may have been displacement through these routes into Spain and Greece, although the magnitude of displacement is much lower than the reduction from Italy’s intervention.

Second, even though fewer migrants are crossing the Mediterranean, those migrants who continue their journey during this period are relying on relatively lower quality smugglers and traveling in more dangerous seas. Panel (d) compares the arrivals and deaths before and after Italy’s intervention. The number of deaths tracks the number of migrants consistently (note the different scales). While there is one peak in deaths in November 2017, it is not abnormal for the winter when few migrants cross and conditions are more treacherous.

At the time of Italy’s intervention in Libya’s port cities, no other policies were being introduced to reduce the flow of migrants out of sub-Saharan Africa and into Libya. Indeed, IOM’s estimates of the number of international migrants in Libya in June, July and August 2017 remained stable. The estimated the number of migrants in May (Round 10), before the intervention, to be 393,652. In September (Round 13), the estimate was 416,556. This jump is comparable to jumps earlier in the year [35]. Events that occurred *after* the Italian intervention, however, may complicate our empirical design. In particular, the battle of Sabratha, which began September 15 and 16 [36], disrupted the major smuggling hub for more than 3 weeks. It is important to demonstrate that the effect we estimate was present before the onset of this battle. We do this in S1 Fig, where we omit the post-battle periods of our sample. The estimated effect is slightly larger in magnitude, suggesting that the reduction in migrants may have been largest right after the intervention. The point estimates from the main analysis and this subsample, however, are not statistically different from one another. Importantly, it is not possible for us to rigorously assess out how events like the battle of Sabratha may have impacted the duration of the effects we estimate. It is plausible that the Italian intervention may have had a lasting effect even in the absence of this port disruption. However, we lack the data needed to more fully explore this possibility.

Overall, Italy’s intervention reduced the number of migrant crossings and deaths in Mediterranean Sea. The event study demonstrates how militias that disengage from smuggling and participate in anti-smuggling efforts can influence the flow of migrants into Europe.

## Robustness

In this section, we consider several challenges to the research design and our interpretation of the influence of political and environments risks on migrant arrivals. We generally find that our results, and the substantive interpretation of them, are robust to possible sources of bias. One natural concern is the exogeneity of political unrest. While sea conditions are plausibly exogenous to migration, it is possible that riots may be triggered by migration and smuggling activity. Another concerns is that riots and migrant crossings share a common cause. Riots may be correlated with port closures, which directly impact smuggling activity. Alternatively economic upheaval may drive riots and create the impetus for migrants to seek out a smuggler to travel across the Mediterranean. We consider these theoretically motivated concerns and find statistically similar results.

We begin by systematically categorizing all the riot events analyzed in our study. ACLED, the source of our data, provides a description of each event. None of the events in our sample are directly linked to international migrants or human smuggling. This is reassuring as the most direct potential source of bias—that migrants or smugglers are triggering or participating in riots—can be ruled out. We then categorize whether a riot event was triggered by or involved internally displaced persons, involved fuel smuggling, or disrupted or closed major ports. We exclude these events from our analysis and replicate the benchmark model from Table 1 in S5 Table. The estimates in Columns 2 through 4, where we exclude these categories of riots, are statistically indistinguishable from the estimate from our preferred specification (Column 1). Economic conditions may influence riots and migration jointly. We conduct a review of the descriptions and find that most of the riots in Libya, where an estimated 90% of Italian sea arrivals originate [3], are political or social in nature. Tunisia, however, experienced economic disruption during the sample period, particularly among workers and merchants. This triggered a number of protests and riots. To account for this concern, we exclude all Tunisian events from our sample in Column 5. The estimate is also indistinguishable from the estimate in our preferred specification.

Another source of potential bias is that sea conditions may influence when riots occur. We investigate this possibility empirically and find little evidence that sea conditions impact riots. An expanding literature in climate economics suggests that climate and weather can drive violence [37, 38]. In our main analysis, we account for any covariance between these two types of risk. Still, there may be concerns about severe multicollinearity. S2 Fig displays a local polynomial regression fit to the unconditional bivariate correlation between riot intensity and wave height. The estimated relationship (solid black line) is nearly flat and centered at the sample mean of riot intensity (dashed red line). This suggests that the correlation between these two factors is nearly zero. In S6 Table we consider a number of alternative models. First, we replicate the benchmark specification from the main analysis (a one week moving average of wave height; one week moving total of riot intensity) and find no unconditional correlation (Column 1) or correlation conditional on month and day-of-week fixed effects (Column 2). Second, we replicate the local polynomial regression using an unconditional and a fixed effects approach in Columns 3 and 4. We then attempt to estimate any lagged effects of wave height on riot intensity in Columns 5 and 6. Across these specifications, we find no statistically significant evidence of any relationship between sea conditions and unrest. Last, it is also possible that sea conditions meaningfully influence the relationship between riots and arrivals. We explore this in S7 Table and find no statistically precise marginal effects.

Taken together, these results suggest that prominent potential sources of bias do not drive the association between riots and migrant arrivals in Italy. Sea conditions are arguably exogenous, but riots are not. Therefore, we are careful to interpret the impact of risky seas on migrant arrivals as causal and the relationship between riots and arrivals as an adjusted correlation. There is substantial evidence of a robust relationship between riots in North Africa and migrant arrivals in Italy. Nevertheless, we note that our research design does not exploit a distinct source of plausibly exogenous variation in unrest, and potential sources of bias due to omitted factors may still complicate a causal interpretation of these results.

Our modeling approach, which leverages log transformations, may be sensitive to alternative transformations. We consider this in S8 Table, where we implement an inverse hyperbolic sine (IHS) transformation. This transformation allows us to limit the potential impact of extreme outliers while avoiding the constant added to the log transformation of riot intensity. Both the estimate on riot intensity and the effect of wave height remain large and statistically precise. These results suggest our preferred estimates are not sensitive to the choice of non-linear transformation used.

Sea conditions may be easily forecasted, which could complicate our empirical design. If migration is driven by future forecasts of poor sea conditions, this may cause a surge in arrivals ahead of bad conditions. Dangerous waves conditions may delay migration, which would cause an uptick in activity after storms. Although each of these changes in migration are driven by environmental risk (through the mechanism we suggest), this may lead us to overstate the impact of wave height since our estimated effects add these changes in movement together.

We evaluate possible bias due to forecasting in several ways and do no find evidence of significant bias. First, we attempt to smooth these shocks to movement by using a three day moving average of arrivals in Italy. These results are presented in S9 Table. Using the moving average yields effects consistent with the main effects (and slightly larger in magnitude). Second, we attempt to estimate the lagged structure of sea conditions on arrivals using leads and lags. By comparing the main estimated effects when we include/exclude the forecasting effect (leads) and delayed surge (long lags), we benchmark the main effect and test for potential bias in the econometric design. We do this in S3 Fig. We observe some suggestive evidence of forecasting and delays, though no systematic patterns across alternative specifications. The main effects are consistent when we compare models with leads and all lags (open circle), all leads (solid circle), and main lags (i.e., the main effects; cross symbol). Thus, our main effects are unlikely to be significantly biased by these mechanisms.

We also revisit the lagged effects of sea conditions on migrant deaths. In the main specification, we sum arrivals and reported deaths on the same day. In S10 Table we lag the reported deaths by one, two, and three days (Columns 2-4 respectively). The estimated effects of wave height are consistent, though they slightly increase in magnitude with additional lags. These results are consistent with the evidence in S4 Table discussed above.

Last, we evaluate the attenuation bias described earlier, and find that our specification underestimates the effect of wave height on migrant arrivals. If relatively few migrants have long journeys (5-7 days), we anticipate more attenuation of the wave height effect relative to attenuation of the coefficient on riot intensity. This is because using our primary approach (7 day average) relatively few migrants would be classified as exposed to future riots (riots that occur after they depart) while most would be classified as exposed to past sea conditions (sea conditions that would have been relevant to earlier migration). We can explore this in the data by comparing the estimates using the prior seven days (our main specification) and the prior three days. We do this in S11 Table. Comparing Columns 1 and 2 suggests attenuation for the coefficient on riot is approximately 12% while attenuation for the wave height effect is 26%. Columns 3 and 4 suggest the riot coefficient’s attenuation is closer to 20% while the attenuation for sea conditions is nearly 30%. For the riots, the alternative lagged relationship is statistically indistinguishable at conventional levels. For wave heights, the differences are large in magnitude and close to statistically precise at the 95% level. Overall, these suggest that our main specification does underestimate the impact of environmental risks on migration.

## Discussion

Our research clarifies how environmental and political risks influence the flow of migrants across the Mediterranean into Italy. An additional study of an intervention in Libya suggests that disrupting smuggling network, by co-opting potential smugglers and increasing the likelihood that migrants are turned around, reduces the flow of arrivals. Taken together, our findings make an important contribution. They highlight how environmental and political risks in transit countries and along the migration path influence patterns of migration and human smuggling.

Migrants crossing the Mediterranean is among the most pressing issues facing the EU. Flows of migrants across the Mediterranean between 2014 and 2019 are among the largest migrant flows into Europe since the EU’s inception. The shear magnitude of the flows and deaths of migrants makes understanding migration in the Mediterranean important for policy and political science scholars. Some of our insights carry over to other contexts too. Previous research on the US-Mexico border has demonstrated that concentrated border enforcement leads to displacement through alternative paths [10]. Smugglers facilitate this displacement, and demand for smuggling services may increase [6]. Our results suggest that migrants respond to risks along their migration path. The context of transit matters for migration and migration policy. Violence in a migrant’s place of residence may encourage a migration. The availability of smuggling networks can make this next move possible.

## Supporting information

S1 Table Summary statistics for time series analysis. (PDF)

S2 Table Alternative types of violence, sea conditions, and migrant flows to Italy. (PDF)

S3 Table Alternative clustering specifications to capture potential temporal autocorrelation in migration to Italy. (PDF)

S4 Table Impact of sea conditions on death rates in Mediterranean Sea. (PDF)

S5 Table Impact of excluding various types of potentially endogenous riot activity on migrant flows to Italy. (PDF)

S6 Table Correlation between sea conditions and riot activity. (PDF)

S7 Table Evaluating the interaction of riots and sea conditions on migrant flows to Italy. (PDF)

S8 Table Using alternative transformation (inverse hyperbolic sine) to evaluate relationships among riots, sea conditions and migrant flows to Italy. (PDF)

S9 Table Evaluating relationships among riots, sea conditions and migration using moving average of arrivals. (PDF)

S10 Table Alternative specifications to capture relationship between total migration (arrivals and deaths/missing) and riots and sea conditions. (PDF)

S11 Table Riots, sea conditions, and migrant flows to Italy using varying lags. (PDF)

S1 Fig Impact of Italian intervention, accounting for post-intervention Battle of Sabratha. Regression-based estimates of the reduction in migrant flows following the intervention, relative the predicted flows from the prior year. Reduction is equivalent to 0.7 standard deviations. Outcome mean is -387.1; standard deviation is 595.2. 95% confidence intervals shown.
(TIF)

S2 Fig Non-parametric local regression between sea conditions and riot activity. Local polynomial regression estimates are reported with 95% confidence intervals in the main plot. The fit line is also plotted. A red dashed line indicates the sample mean for riot activity by day (0.1725). A histogram of wave height is reported below the main plot.
(TIF)

S3 Fig Using leads and lags of sea conditions to calibrate main effects for forecasting (anticipation) and departure delays. Time periods vary by subplot (daily bins; three-day bins; seven-day bins) and are noted in each axis title.
(TIF)
