# Supplementary material for: Political and environmental risks influence migration and human smuggling across the Mediterranean Sea
Source: PLoS One. 2020 Jul 31;15(7):e0236646. doi: 10.1371/journal.pone.0236646 (PMC7394383; doi:10.1371/journal.pone.0236646)
Supplement: S1 Table — (PDF) [file pone.0236646.s001.pdf]

Variable	Mean	Std. Dev.	Min.	Max.	N
Arrivals	378.148	738.546	0	5504	812
Arrivals, ln	3.282	2.961	0	8.613	812
Arrivals+Deaths, ln	3.611	2.749	0	8.614	812
Arrivals+Deaths, ln (1 day lag of Deaths)	3.594	2.769	0	8.618	812
Arrivals+Deaths, ln (2 day lag of Deaths)	3.569	2.787	0	8.622	812
Arrivals+Deaths, ln (3 day lag of Deaths)	3.618	2.762	0	8.613	812
Arrivals, IHS	3.688	3.273	0	9.306	812
Arrivals+Deaths, IHS	4.087	3.012	0	9.307	812
Arrivals, ln 3 day avg	4.42	2.34	0	8.218	812
Arrivals+Deaths, ln 3 day avg	4.641	2.068	0	8.218	812
Death/Missing Rate	0.244	0.407	0	1	618
Riots, prior week total	1.929	1.757	0	8	812
Riots, ln prior week total	0.91	0.579	0	2.197	812
Riots, IHS prior week avg	1.179	0.751	0	2.776	812
Riots, ln daily	0.173	0.337	0	1.792	812
Wave Height, prior week avg	1.088	0.412	0.327	2.282	812
Wave Height, ln prior week avg	0.01	0.393	-1.119	0.825	812
Wave Height, IHS prior week avg	0.915	0.27	0.321	1.563	812
Wave Height, ln daily	-0.066	0.544	-1.43	1.549	812
Wave Height, ln days 1-3 future avg	-0.028	0.476	-1.204	1.236	812
Wave Height, ln days 1-3 prior avg	-0.026	0.478	-1.204	1.236	812
Wave Height, ln days 4-6 prior avg	-0.027	0.477	-1.204	1.236	812

S1 Table. Summary statistics for time series analysis
