# Supplementary material for: Political and environmental risks influence migration and human smuggling across the Mediterranean Sea
Source: PLoS One. 2020 Jul 31;15(7):e0236646. doi: 10.1371/journal.pone.0236646 (PMC7394383; doi:10.1371/journal.pone.0236646)
Supplement: S2 Table — (PDF) [file pone.0236646.s002.pdf]

	(1)	(2)	(3)	(4)
Riots (ln, prior week total)	0.503**			0.411*
	(0.200)			(0.220)
Violence Against Local Civilians (ln, prior week total)		0.439*		0.265
		(0.260)		(0.289)
Rebel-Government Violence (ln, prior week total)			0.0793	0.00240
			(0.179)	(0.171)
Wave Height (ln, prior week average)	-2.542***	-2.533***	-2.556***	-2.522***
	(0.364)	(0.362)	(0.368)	(0.368)
Number of Observations	812	812	812	812
R^2	0.0802	0.0762	0.0701	0.0822

Notes: Outcome of interest is the daily total of migrants arriving in Italy (ln) (Columns 1-4). Driscoll-Kraay temporal autocorrelation robust standard errors (clustered by 14 day windows) are reported. Various columns introduce supplemental measures of potential violence exposure. Rows one and two (riots and violence against civilians) are not statistically distinct (test of equality yields $p = 0.7351$). Stars indicate *** $p < 0.01$, ** $p < 0.05$, * $p < 0.1$.

S2 Table. Alternative types of violence, sea conditions, and migrant flows to Italy
