# Supplementary material for: Political and environmental risks influence migration and human smuggling across the Mediterranean Sea
Source: PLoS One. 2020 Jul 31;15(7):e0236646. doi: 10.1371/journal.pone.0236646 (PMC7394383; doi:10.1371/journal.pone.0236646)
Supplement: S3 Table — (PDF) [file pone.0236646.s003.pdf]

	(1)	(2)	(3)	(4)
Riots (ln, prior week total)	0.503**	0.503**	0.503**	0.503**
	(0.199)	(0.199)	(0.206)	(0.191)
Wave Height (ln, prior week average)	-2.542***	-2.542***	-2.542***	-2.542***
	(0.363)	(0.358)	(0.316)	(0.335)
Number of Observations	812	812	812	812
R^2	0.0802	0.0802	0.0802	0.0802

Notes: Outcome of interest is the daily total of migrants arriving in Italy (ln) (Columns 1-4). Driscoll-Kraay temporal autocorrelation robust standard errors (clustered by varying day windows) are reported in Columns 1-3. Column 1 uses a 21 day window; Column 2 uses a 28 day window; Column 3 uses a 56 day window. Column 4 clusters standard errors by calendar month. Stars indicate *** $p < 0.01$, ** $p < 0.05$, * $p < 0.1$.

S3 Table. Alternative clustering specifications to capture potential temporal autocorrelation in migration to Italy
