# Supplementary material for: Political and environmental risks influence migration and human smuggling across the Mediterranean Sea
Source: PLoS One. 2020 Jul 31;15(7):e0236646. doi: 10.1371/journal.pone.0236646 (PMC7394383; doi:10.1371/journal.pone.0236646)
Supplement: S4 Table — (PDF) [file pone.0236646.s004.pdf]

	(1)	(2)	(3)	(4)	(5)
Wave Height (ln, current)	0.167***		0.0528		
	(0.0415)		(0.0473)		
Wave Height (ln, lag 1)		0.252***	0.154***		
		(0.0381)	(0.0501)		
Wave Height (ln, lag 2)			0.0828		
			(0.0575)		
Wave Height (ln, lag 3)			0.0504		
			(0.0593)		
Wave Height (ln, lag 4)			0.0429		
			(0.0536)		
Wave Height (ln, lag 5)			-0.0529		
			(0.0472)		
Wave Height (ln, lag 6)			0.0388		
			(0.0402)		
Wave Height (ln, previous 3 days)				0.309***	
				(0.0470)	
Wave Height (ln, prior week average)					0.266***
					(0.0480)
Number of Observations	618	617	615	618	618
R^2	0.0421	0.0889	0.111	0.0973	0.0388

Notes: Outcome of interest is the daily total of migrants arriving in Italy (ln) (Columns 1-4). Driscoll-Kraay temporal autocorrelation robust standard errors (clustered by 14 day windows) are reported. Stars indicate *** $p < 0.01$, ** $p < 0.05$, * $p < 0.1$.

S4 Table. Impact of sea conditions on death rates in Mediterranean Sea
