# Supplementary material for: Political and environmental risks influence migration and human smuggling across the Mediterranean Sea
Source: PLoS One. 2020 Jul 31;15(7):e0236646. doi: 10.1371/journal.pone.0236646 (PMC7394383; doi:10.1371/journal.pone.0236646)
Supplement: S5 Table — (PDF) [file pone.0236646.s005.pdf]

	(1)	(2)	(3)	(4)	(5)
Riots (ln, prior week total)	0.503**				
	(0.200)				
Riots (ln, excluding IDP related events)		0.474**			
		(0.207)			
Riots (ln, excluding fuel smuggling related events)			0.484**		
			(0.201)		
Riots (ln, excluding port closure related events)				0.480**	
				(0.200)	
Riots (ln, excluding economic riots in Tunisia)					0.519**
					(0.262)
Wave Height (ln, prior week average)	-2.542***	-2.525***	-2.557***	-2.546***	-2.555***
	(0.364)	(0.364)	(0.365)	(0.365)	(0.376)
Number of Observations	812	812	812	812	812
R^2	0.0802	0.0795	0.0793	0.0792	0.0770

Notes: Outcome of interest is the daily total of migrants arriving in Italy (ln) (Columns 1-5). Driscoll-Kraay temporal autocorrelation robust standard errors (clustered by 14 day windows) are reported. Additional rows capture alternative measures of riot activity, excluding subsets of events from the total sample of riots. Stars indicate *** $p < 0.01$, ** $p < 0.05$, * $p < 0.1$.

S5 Table. Impact of excluding various types of potentially endogenous riot activity on migrant flows to Italy
