# Supplementary material for: Political and environmental risks influence migration and human smuggling across the Mediterranean Sea
Source: PLoS One. 2020 Jul 31;15(7):e0236646. doi: 10.1371/journal.pone.0236646 (PMC7394383; doi:10.1371/journal.pone.0236646)
Supplement: S6 Table — (PDF) [file pone.0236646.s006.pdf]

	(1)	(2)	(3)	(4)	(5)	(6)
Wave Height (ln, prior week average)	-0.00838	-0.0667				
	(0.0487)	(0.142)				
Wave Height (ln, current)			-0.0106	-0.0211	-0.0113	-0.0174
			(0.0211)	(0.0250)	(0.0324)	(0.0304)
Wave Height (ln, lag 1)					-0.0251	-0.0221
					(0.0400)	(0.0389)
Wave Height (ln, lag 2)					0.0331	0.0291
					(0.0423)	(0.0399)
Wave Height (ln, lag 3)					0.00842	0.0110
					(0.0410)	(0.0417)
Wave Height (ln, lag 4)					-0.0191	-0.0244
					(0.0431)	(0.0405)
Wave Height (ln, lag 5)					0.0398	0.0414
					(0.0433)	(0.0434)
Wave Height (ln, lag 6)					-0.0151	-0.0162
					(0.0314)	(0.0314)
Number of Observations	812	812	812	812	806	806
R^2	0.0000324	0.00114	0.000293	0.000917	0.00391	0.00406

Notes: Outcome of interest is the weekly total of riot activity (ln) in Columns 1 and 2. In Columns 3-6, outcome is daily total of riot activity (ln). Unconditional correlations are reported in odd numbered columns; regression with month and day-of-week fixed effects are reported in even numbered columns. Robust standard errors are reported in odd numbered columns; Driscoll-Kraay temporal autocorrelation robust standard errors (clustered by 14 day windows) are reported in even numbered columns. Stars indicate *** $p < 0.01$, ** $p < 0.05$, * $p < 0.1$.

S6 Table. Correlation between sea conditions and riot activity
