# Supplementary material for: Political and environmental risks influence migration and human smuggling across the Mediterranean Sea
Source: PLoS One. 2020 Jul 31;15(7):e0236646. doi: 10.1371/journal.pone.0236646 (PMC7394383; doi:10.1371/journal.pone.0236646)
Supplement: S7 Table — (PDF) [file pone.0236646.s007.pdf]

	(1)	(2)	(3)	(4)
Riots (ln, prior week total)	0.503**	0.499**	0.467**	0.469**
	(0.200)	(0.205)	(0.197)	(0.195)
Wave Height (ln, prior week average)	-2.542***	-2.597***	-2.259***	-2.225***
	(0.364)	(0.606)	(0.352)	(0.563)
Riots (ln, prior week total) × Wave Height (ln, prior week average)		0.0668		-0.0419
		(0.529)		(0.492)
Number of Observations	812	812	812	812
R^2	0.0802	0.0802	0.0742	0.0742

Notes: Outcome of interest is the daily total of migrants arriving in Italy (ln) (Columns 1-2); Columns 3 and 4 present evidence where the outcome is arrivals and missing migrants (ln). Driscoll-Kraay temporal autocorrelation robust standard errors (clustered by 14 day windows) are reported. Stars indicate *** $p < 0.01$, ** $p < 0.05$, * $p < 0.1$.

S7 Table. Evaluating the interaction of riots and sea conditions on migrant flows to Italy
