# Supplementary material for: Political and environmental risks influence migration and human smuggling across the Mediterranean Sea
Source: PLoS One. 2020 Jul 31;15(7):e0236646. doi: 10.1371/journal.pone.0236646 (PMC7394383; doi:10.1371/journal.pone.0236646)
Supplement: S8 Table — (PDF) [file pone.0236646.s008.pdf]

	(1)	(2)	(3)	(4)
Riots (ln, prior week total)	0.503**		0.467**	
	(0.200)		(0.197)	
Riots (inverse hyperbolic sine, prior week total)		0.394**		0.366**
		(0.169)		(0.166)
Wave Height (ln, prior week average)	-2.542***		-2.259***	
	(0.364)		(0.352)	
Wave Height (inverse hyperbolic sine, prior week average)		-4.118***		-3.576***
		(0.538)		(0.544)
Number of Observations	812	812	812	812
R^2	0.0802	0.0810	0.0742	0.0732

Notes: Outcome of interest is the daily total of migrants arriving in Italy (ln) (Column 1) and the sum of arrivals and missing migrants (Column 3). Outcome of interest in Columns 2 is the daily total of migrants arriving in Italy transformed using the inverse sine hyperbolic function; Column 4 presents the comparable transformation of arrivals and missing migrants. Relevant changesare made to each Driscoll-Kraay temporal autocorrelation robust standard errors (clustered by 14 day windows) are reported. Stars indicate *** $p < 0.01$, ** $p < 0.05$, * $p < 0.1$.

S8 **Table.** Using alternative transformation (inverse hyperbolic sine) to evaluate relationships among riots, sea conditions and migrant flows to Italy
