# Supplementary material for: Political and environmental risks influence migration and human smuggling across the Mediterranean Sea
Source: PLoS One. 2020 Jul 31;15(7):e0236646. doi: 10.1371/journal.pone.0236646 (PMC7394383; doi:10.1371/journal.pone.0236646)
Supplement: S9 Table — (PDF) [file pone.0236646.s009.pdf]

	(1)	(2)
Riots (ln, prior week total)	0.532***	0.494***
	(0.186)	(0.176)
Wave Height (ln, prior week average)	-3.152***	-2.749***
	(0.324)	(0.293)
Number of Observations	812	812
R^2	0.201	0.199

Notes: Outcome of interest is the three day moving average of migrants arriving in Italy (ln) (Column 1) and the three day moving average for arrivals and deaths (ln) (Column 2). Driscoll-Kraay temporal autocorrelation robust standard errors (clustered by 14 day windows) are reported. Stars indicate *** $p < 0.01$, ** $p < 0.05$, * $p < 0.1$.

S9 Table. Evaluating relationships among riots, sea conditions and migration using moving average of arrivals
