# Supplementary material for: Political and environmental risks influence migration and human smuggling across the Mediterranean Sea
Source: PLoS One. 2020 Jul 31;15(7):e0236646. doi: 10.1371/journal.pone.0236646 (PMC7394383; doi:10.1371/journal.pone.0236646)
Supplement: S10 Table — (PDF) [file pone.0236646.s010.pdf]

	(1)	(2)	(3)	(4)
Riots (ln, prior week total)	0.467**	0.457**	0.465**	0.476**
	(0.197)	(0.192)	(0.199)	(0.187)
Wave Height (ln, prior week average)	-2.259***	-2.508***	-2.459***	-2.623***
	(0.352)	(0.352)	(0.348)	(0.357)
Number of Observations	812	812	812	812
R^2	0.0742	0.0862	0.0831	0.0953

Notes: Outcome of interest is the daily total of migrants arriving in Italy and reported missing migrants (deaths) (ln). In Column 1, this measures are calculated contemporaneously (same day). In Column 2, we lag the missing migrants by one day; Column 3 by two days; Column 4 by three days. This lags account for possibility that migrants would have arrived up to three days after they were discovered at sea or reported missing. Driscoll-Kraay temporal autocorrelation robust standard errors (clustered by 14 day windows) are reported. Stars indicate *** $p < 0.01$, ** $p < 0.05$, * $p < 0.1$.

S10 Table. Alternative specifications to capture relationship between total migration (arrivals and deaths/missing) and riots and sea conditions
