# Supplementary material for: Political and environmental risks influence migration and human smuggling across the Mediterranean Sea
Source: PLoS One. 2020 Jul 31;15(7):e0236646. doi: 10.1371/journal.pone.0236646 (PMC7394383; doi:10.1371/journal.pone.0236646)
Supplement: S11 Table — (PDF) [file pone.0236646.s011.pdf]

	(1)	(2)	(3)	(4)
Riots (ln, prior week total)	0.503**		0.467**	
	(0.200)		(0.197)	
Riots (ln, prior 3 days total)		0.567***		0.564***
		(0.182)		(0.172)
Wave Height (ln, prior week average)	-2.542***		-2.259***	
	(0.364)		(0.352)	
Wave Height (ln, prior 3 days average)		-3.210***		-2.918***
		(0.242)		(0.234)
Number of Observations	812	812	812	812
R^2	0.0802	0.222	0.0742	0.215

Notes: Outcome is the daily total of migrants arriving in Italy (ln) (Columns 1-2). In Column 3 and 4, the outcome is the daily total flow of migrants (the sum of arrivals and reported deaths and disappearances). Driscoll-Kraay temporal autocorrelation robust standard errors (clustered by 14 day windows) are reported. Stars indicate *** $p < 0.01$, ** $p < 0.05$, * $p < 0.1$.

S11 Table. Riots, sea conditions, and migrant flows to Italy using varying lags
