# Supplementary figures and images for: Political and environmental risks influence migration and human smuggling across the Mediterranean Sea
Source: PLoS One. 2020 Jul 31;15(7):e0236646. doi: 10.1371/journal.pone.0236646 (PMC7394383; doi:10.1371/journal.pone.0236646)

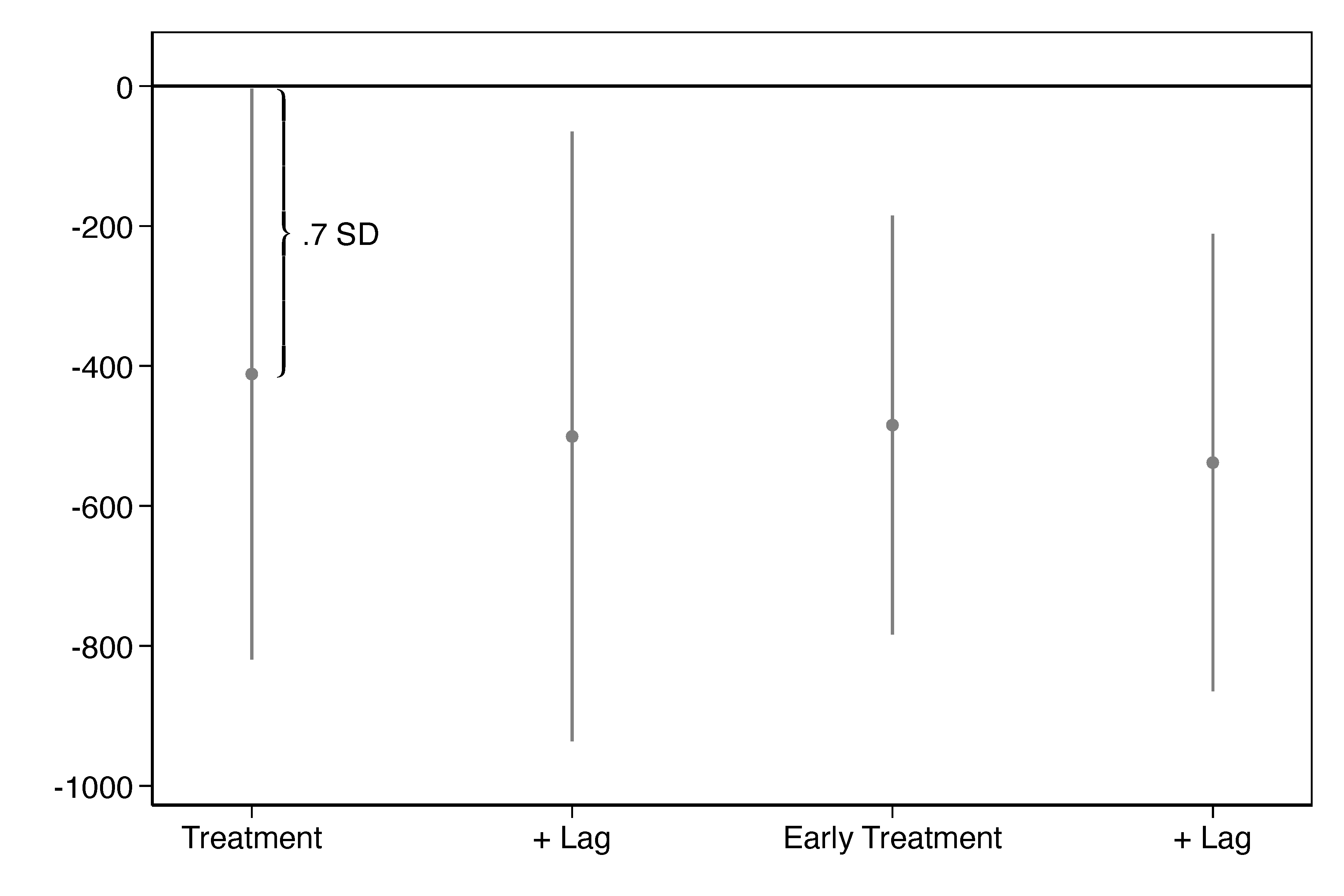

Supplement: S1 Fig — Regression-based estimates of the reduction in migrant flows following the intervention, relative the predicted flows from the prior year. Reduction is equivalent to 0.7 standard deviations. Outcome mean is -387.1; standard deviation is 595.2. 95% confidence intervals shown. (TIF) [file pone.0236646.s012.tif]

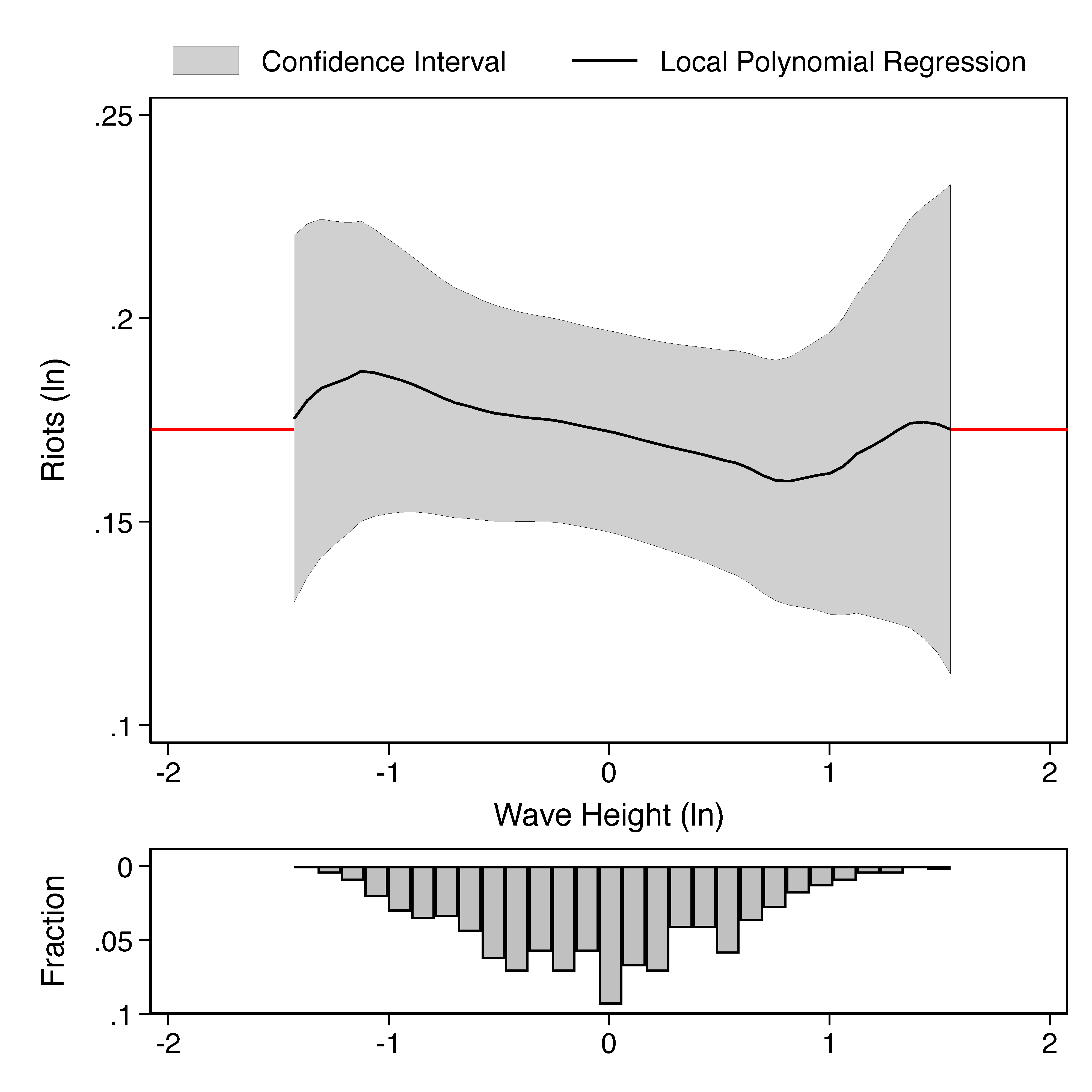

Supplement: S2 Fig — Local polynomial regression estimates are reported with 95% confidence intervals in the main plot. The fit line is also plotted. A red dashed line indicates the sample mean for riot activity by day (0.1725). A histogram of wave height is reported below the main plot. (TIF) [file pone.0236646.s013.tif]

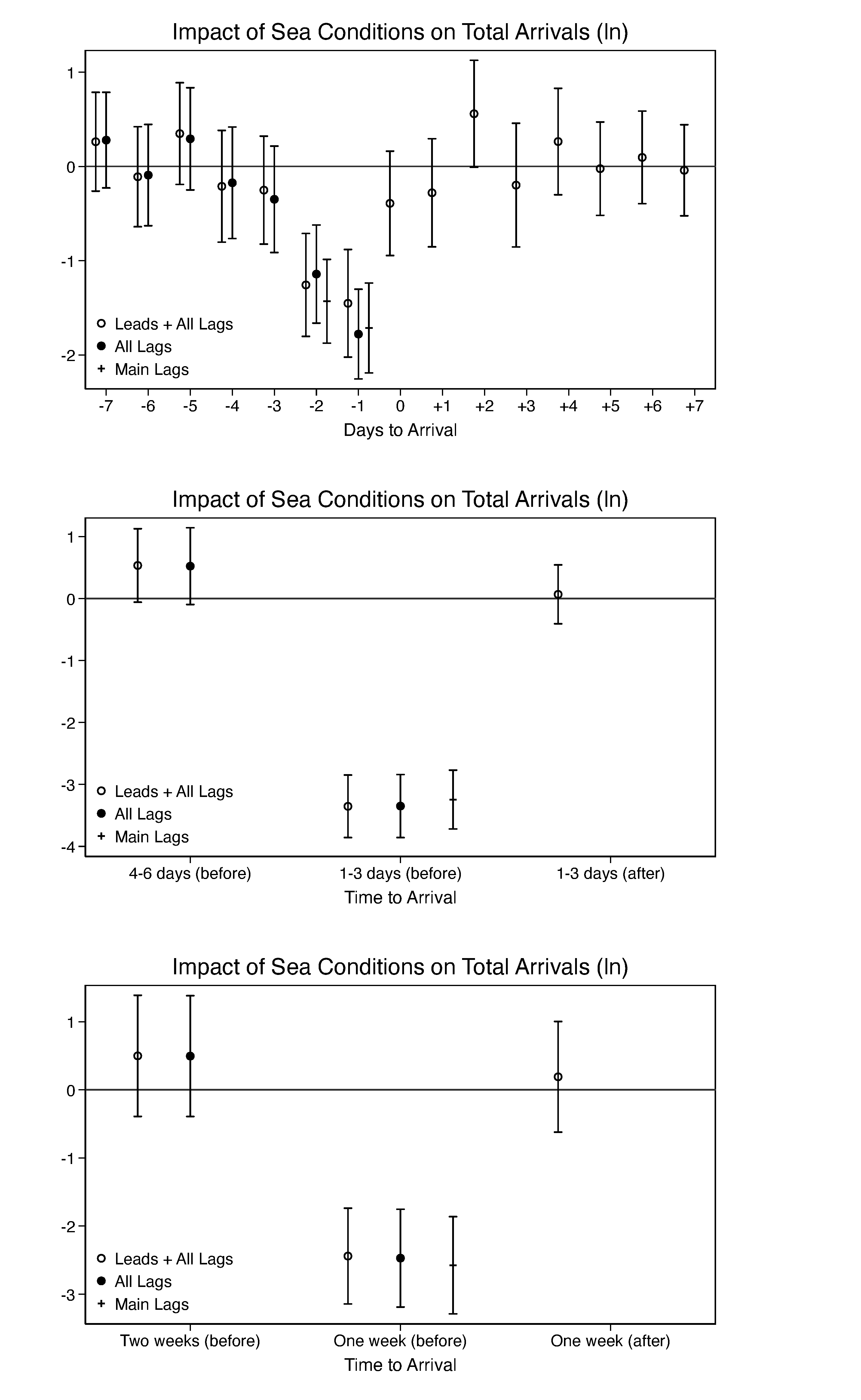

Supplement: S3 Fig — Time periods vary by subplot (daily bins; three-day bins; seven-day bins) and are noted in each axis title. (TIF) [file pone.0236646.s014.tif]
